# CRISPR Inhibition of Prophage Acquisition in *Streptococcus pyogenes*

**DOI:** 10.1371/journal.pone.0019543

**Published:** 2011-05-06

**Authors:** Takashi Nozawa, Nayuta Furukawa, Chihiro Aikawa, Takayasu Watanabe, Bijaya Haobam, Ken Kurokawa, Fumito Maruyama, Ichiro Nakagawa

**Affiliations:** 1 Section of Bacterial Pathogenesis, Graduate School of Medical and Dental Sciences, Tokyo Medical and Dental University, Tokyo, Japan; 2 Department of Medical Genome Sciences, Graduate School of Frontier Sciences, The University of Tokyo, Kashiwa, Japan; 3 Division of Information Biotechnology, Department of Bioinformation Engineering, Tokyo Institute of Technology School and Graduate School of Bioscience and Biotechnology, Yokohama, Japan; Instituto Butantan, Brazil

## Abstract

*Streptococcus pyogenes*, one of the major human pathogens, is a unique species since it has acquired diverse strain-specific virulence properties mainly through the acquisition of streptococcal prophages. In addition, *S. pyogenes* possesses clustered regularly interspaced short palindromic repeats (CRISPR)/Cas systems that can restrict horizontal gene transfer (HGT) including phage insertion. Therefore, it was of interest to examine the relationship between CRISPR and acquisition of prophages in *S. pyogenes*. Although two distinct CRISPR loci were found in *S. pyogenes*, some strains lacked CRISPR and these strains possess significantly more prophages than CRISPR harboring strains. We also found that the number of spacers of *S. pyogenes* CRISPR was less than for other streptococci. The demonstrated spacer contents, however, suggested that the CRISPR appear to limit phage insertions. In addition, we found a significant inverse correlation between the number of spacers and prophages in *S. pyogenes*. It was therefore suggested that *S. pyogenes* CRISPR have permitted phage insertion by lacking its own spacers. Interestingly, in two closely related *S. pyogenes* strains (SSI-1 and MGAS315), CRISPR activity appeared to be impaired following the insertion of phage genomes into the repeat sequences. Detailed analysis of this prophage insertion site suggested that MGAS315 is the ancestral strain of SSI-1. As a result of analysis of 35 additional streptococcal genomes, it was suggested that the influences of the CRISPR on the phage insertion vary among species even within the same genus. Our results suggested that limitations in CRISPR content could explain the characteristic acquisition of prophages and might contribute to strain-specific pathogenesis in *S. pyogenes*.

## Introduction

During evolution, bacteria acquired new traits primarily by horizontal gene transfer (HGT) as a key driving force for expressing novel pathogenic properties, new colonization niches as well as metabolic adaptations [1], [2], [3], [4], [5]. Conjugation, transduction and transformation are the major mechanisms for HGT. The contributions and the impact of each mechanism are variable among species [6]. Of the three HGT mechanisms, transduction involving bacteriophage-mediated DNA transfer often provides the profound alteration in host bacterial genomes. This process can also convert a non-pathogenic strain into a pathogenic variety through prophage-encoded toxins, surface alterations, or increasing resistance to human immunity [7]. In addition, prophage insertion into the host genome often inactivates or alters the host genes [7], [8].

In contrast, phages can cause lytic infection and phage infection is often a danger to host bacteria [7], [9]. Therefore, phage infection can have divergent effects: new traits acquisition as an advantage and bacteriolysis as a disadvantage. To protect against the invading phages, bacteria have developed several defense mechanisms such as prevention of adsorption, blocking injection, cleaving phage nucleic acid, and aborting infection [10]. Recently, a novel defense system, clustered regularly interspaced short palindromic repeats (CRISPR) loci, has been identified as a form of acquired immunity against invading foreign DNA including bacteriophage and plasmid DNA [11], [12], [13]. CRISPR loci are found in almost all Archaea and approximately 40% of sequenced bacterial genomes. They composed of a short repeat sequence (21–47 bp) separated by a unique variable sequence called a spacer [14], [15], [16]. The repeat sequence is highly conserved within a particular CRISPR locus. In contrast, the spacers vary greatly and their sequences have similarity to phages and plasmids and sometimes to host chromosomal sequences [17]. Each CRISPR is commonly followed by a conserved AT-rich sequence known as a leader sequence. CRISPR-associated (*cas*) genes, essential components of the system, are located adjacent to the CRISPR loci [18]. Acquired immunity involving CRISPR/Cas systems can be divided into two stages: the acquisition stage for uptake of the foreign element as a spacer into the leader-proximal end of CRISPR, and the immunity stage involving interference with the targeting of DNA in a sequence-specific manner [18], [19]. This role was first demonstrated experimentally in a bacterium important in the dairy industry, *Streptococcus thermophilus*, in which CRISPR-harboring strains acquired resistance to infection by phages by incorporating novel spacers derived from the previously infected phages [11], [20]. The CRISPR/Cas system was also reported to limit HGT in other bacteria such as staphylococci [12].

In addition to *S. thermophilus*, there are several other medically and economically important species in the genus Streptococcus such as *S. mutans*, *S. pneumoniae* and *S. pyogenes*. *S. mutans* is known to be principal aetiological agent of dental caries and *S. pneumoniae* is the most common cause of lobar pneumonia. *S. pyogenes* causes a wide range of infections, including pharyngitis, sepsis, toxic shock-like syndrome, and life-threatening necrotizing fasciitis [21]. In all of these species, HGT appears to have played an important role in their evolution. For example, the uptake of exogenous DNA by transformation apparently increased the diversity of *S. pneumoniae* and *S. sanguinis* while insertion sequences (IS) and transposons contribute to the genetic diversity of *S. mutans* [22], [23], [24], [25], [26], [27]. Most notably, *S. pyogenes* is a unique species which has acquired strain-specific virulence genes by means of multiple prophages [28], [29]. The sequences of 13 strain-specific genomes from *S. pyogenes* revealed the existence of 2–8 prophages and ∼90% conserved genomic sequences (excluding exogenous genetic regions) [29], [30] therefore suggesting that their diversity and disease causing capacity might be related to the acquisition of prophages [31]. Because of the predicted role of CRISPR in limiting HGT including phage insertions, it would be of significance to determine whether the CRISPR are involved in the acquisition of prophages in *S. pyogenes*. In this study, we examined 13 sequenced *S. pyogenes* strains and the relationship between CRISPR and the acquisition of prophages. In addition, we extended the analysis of the distribution of CRISPR and prophages by examining a total of 35 streptococcal strain sequences.

## Results

### *S. pyogenes* has two distinct CRISPR loci containing relatively few spacers

We determined the distributions of CRISPR loci and *cas* genes for all of the 13 sequenced *S. pyogenes* strains. 15 CRISPR were found and classified as two distinct loci (designated CRISPR1 and CRISPR2) based on their typical repeat sequences. Of the 13 strains, seven strains (SF370, MGAS5005, MGAS10270, MGAS2096, MGAS9429, MGAS6180, NZ131) had both CRISPR1 and CRISPR2, one strain (MGAS10750) possessed only the CRISPR2 locus, and the position of each one of the CRISPR loci was conserved across strains (Table 1, Fig. S1). In contrast, five strains (MGAS315, SSI-1, MGAS8232, Manfredo, MGAS10394) had no CRISPR loci. The typical repeat sequences of both CRISPR1 and CRISPR2 were highly conserved between the strains (Table S1). The typical repeat sequences of CRISPR1 and CRISPR2 belong to the repeat clusters 10 and 3 previously defined, respectively [32]. As is often the case, the terminal repeat sequences of CRISPR1 and CRISPR2 are relatively degenerate compared with their typical repeat sequences (Table S1) [20], [33], [34], [35]. CRISPR1 is located between *hemN* and *lepA* while CRISPR2 is positioned between *valS* and *msrA* (Fig. S2). In MGAS10750, MGAS315, and SSI-1, one sequence that is similar to the CRISPR1 terminal repeat sequence but not remainder of CRISPR (i.e. not a cluster of repeat sequences) was found (Table S1). Although the CRISPR1 terminal repeat-like sequence of MGAS10750 is also located between *hemN* and *lepA*, those of MGAS315 and SSI-1 are distant by 39.5 kb from the CRISPR1 locus of the other strains (Fig. S1A, B). This interesting case is described more in detail below.

**Table 1:** Distribution of CRISPR loci and prophages in *S. pyogenes*.

Strain	M type	CRISPR1^a^	CRISPR1 location^b^	CRISPR2^a^	CRISPR2 location^b^	Prophage^c^	ICE^d^
SF370	1	7	1049–1050	4	1559–1561	4	1
MGAS5005	1	4	772–773	5	1284–1285	3	1
MGAS10270	2	3	889–890	4	1364–1365	5	2
MGAS315	3	(1)^e^	889–890	-	-	6	0
SSI-1	3	(1)^e^	937–737	-	-	6	0
MGAS10750	4	(1)^e^	736–737	6	1391–1392	4	2
Manfredo	5	-	-	-	-	5	0
MGAS10394	6	-	-	-	-	8	0
MGAS2096	12	3	846–847	7	1303–1304	2	2
MGAS9429	12	3	888–889	8	1278–1279	3	1
MGAS8232	18	-	-	-	-	5	0
MGAS6180	28	5	751–752	2	1288–1289	4	3
NZ131	49	5	827–828	6	1206–1207	3	0

a The number of repeats are shown.

b Gene numbers that located both side of CRISPR are shown.

c The number of prophage regions are shown.

d The number of ICE are shown.

e (1) indicate that the presence of one terminal repeat-like sequence.

f - indicate the absence of CRISPR locus.

We next identified the *cas* genes in the *S. pyogenes* genome. *cas* gene sets are clustered into 8 subtypes based on the member of *cas* genes [36]. The CRISPR clusters 10 and 3 usually are associated with the Nmeni and Dvulg subtype *cas* genes, respectively [32]. Indeed, Nmeni and Dvulg subtype *cas* genes were found upstream from CRISPR1 and CRISPR2, respectively (Fig. S2). Not only the seven CRISPR1-harboring strains but MGAS10750 and MGAS315 also have Nmeni subtype *cas* genes at the same relative position as CRISPR1-harboring strains. Since the repeat sequence, the *cas* gene subsets and the location of CRISPR/*cas* loci were conserved among the strains, it is suggested that *S. pyogenes* harbored CRISPR/*cas* loci early in their evolution. Therefore, the absence of CRISPR may have been adaptive by allowing the integration of bacteriophages into genomes to acquire new traits like Enterococci, which could acquire antibiotic resistance genes for environmental adaptation [20], [37].

**Figure 1:**
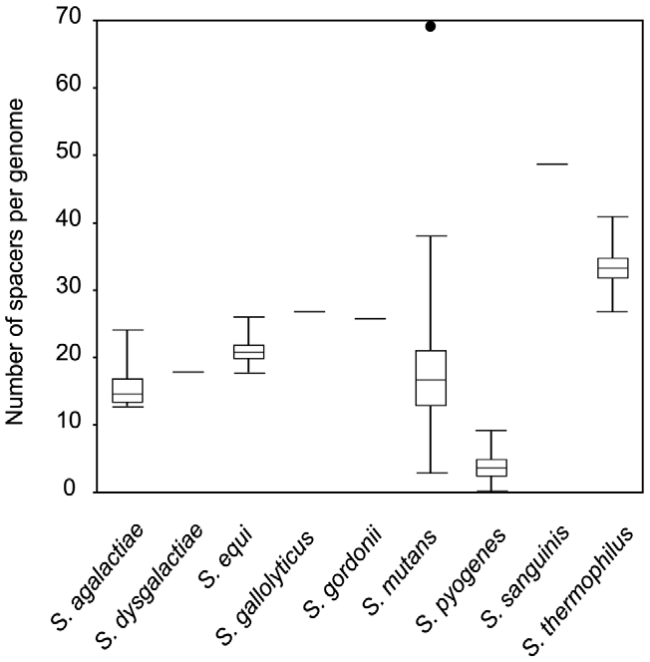
Box plot of the number of spacers in streptococcal CRISPR. The boxes indicate the medians and 25^th^–75^th^ percentiles of the number of spacers per genome of streptococcal CRISPR. Whiskers indicate 5^th^ to 95^th^ percentiles and outliers are indicated by the closed circle.

As CRISPR/*cas* loci are widely distributed among streptococcal species, we compared the characteristics of CRISPR/*cas* among these species. The repeat sequence, repeat size, spacer size, and *cas* genes subtypes are very similar within the same repeat cluster (Table S2). Interestingly, we found a major difference in the number of spacers per locus. The mean number of repeats per genome within *S. pyogenes* was only 6.6, which was significantly fewer than for other streptococci (*P*<0.01) (Fig. 1). Because it was reported that there is a correlation between the numbers of spacers in a CRISPR locus and phage resistance [11], [15], it is suggested that phage resistance in *S. pyogenes* is lower than that for other streptococci.

### Spacer deletion and acquisition in *S. pyogenes* CRISPR

**Figure 2:**
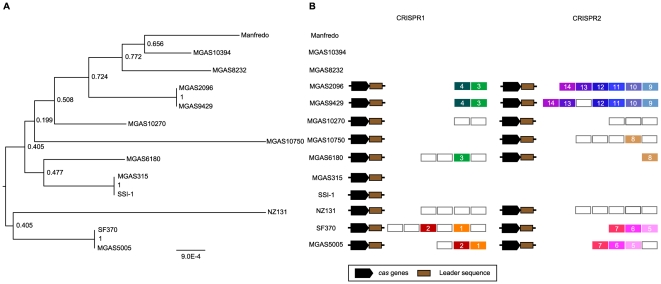
Phylogenetic relationship and spacers across the two CRISPR loci for *S. pyogenes* strains. (A) An MLST-based dendrogram of the 13 strains was generated using the *S. pyogenes* MLST database (see Materials and Methods). (B) Conserved spacers among strains were shown as colored boxes. Single spacers appear in white background; identical spacers are represented using a same color background and identical number. *cas* genes and leader sequences are represented by black and brown boxes, respectively.

Since new spacers are regularly added at the end of the repeat cluster adjoining the leader sequence as a spacer-repeat unit [11], [34], [38], [39], [40], there is regularity in the alignment of the sequence of spacers. It has been established experimentally and computationally that the leader-proximal end of spacers are more diversified and the leader-distal end of spacers are more conserved among strains [20], [41]. Indeed, in *S. mutans* and *S. thermophilus*, similar spacer structures were observed [20], [42]. In contrast, *S. pyogenes* spacer structure was characteristic in that the spacers were variable between strains and the most conserved spacers were observed in only closely related strains (Fig. 2A, B). This suggests that the progenitor spacers were deleted and the existing spacers were obtained relatively recently after their diversification [43]. These observations indicate that both spacer acquisition and deletion are active in *S. pyogenes* CRISPR. Considering the location of deleted spacers, the deletions seem to occur randomly and is consistent with previous reports in *Mycobacterium tuberculosis* and *Yersinia pestis* [39], [44].

Despite the fact that *S. pyogenes* CRISPR appear to be active in spacer acquisition, the number of spacers is small. In the spacer acquisition stage, it is known that Cas1 functions to integrate novel spacers into the CRISPR loci [45]. Hence, small number of *S. pyogenes* CRISPR spacers may result from a mutation or inactivation of Cas1. In light of this, we examined the *cas1* gene sequences in *S. pyogenes*. However, the amino acid sequences of Cas1 were highly conserved (>96% identity) among strains, and the sequences exhibited high similarity with the sequence of *S. thermophilus* LMD-9 (79.6% identity) and *S. mutans* NN2025 (87.15% identity), whose CRISPR are thought to function in acquiring novel spacers [20], [27]. The phylogenic tree for the *cas1* sequence was similar to the MLST tree of *S. pyogenes* suggesting that the CRISPR/*cas* loci were not acquired recently but may have existed in an early ancestor (data not shown).

### *S. pyogenes* CRISPR inhibits phage insertion

To assess the functionality of *S. pyogenes* CRISPR, we examined their spacer sequences and defined their respective protospacers. It was previously shown that perfect identity between spacer and protospacer is required to provide immunity [11], [46]. However, because of the rapid evolution of phage sequences, we compared the protospacers with a criteria of >95% identity. Of 41 distinct spacers, 27 spacers matched streptococcal phage genomes and one spacer matches one of its own chromosomal gene sequences with >95% identity (Table 2). The remaining 13 spacers (32%) did not match known sequences, which may reflect the small number of sequenced phages and the existence of unknown phages which have recently infected these strains. All spacers that matched streptococcal phages showed exact or approximate matches with the sequences of the prophage regions in various *S. pyogenes* strains (Fig. S3). For example, the most recently added spacer (i.e. the spacer adjacent to the leader sequence) in MGAS370 CRISPR1 matched the sequences within the prophage regions of MGAS10394, MGAS6180, MGAS8232 and SSI-1 (Fig. S3). This suggests that *S. pyogenes* has acquired the spacers following infection with similar streptococcal phages. Of note, despite the fact that all 26 spacers derived from streptococcal phages match the variable prophage regions in *S. pyogenes* genomes, there is no spacer which is homologous to a prophage region in its own genome (Table 2). This strongly suggests that *S. pyogenes* CRISPR are antagonistic to phage insertions.

**Table 2:** Characteristics of spacers in *S. pyogenes*.

	CRISPR1	CRISPR2
Spacer size (bp)	30 (30–31)	35 (33–36)
Number of spacers	23	33
Number of distinct spacers	18	23
Number of single spacers	14 (78%)	13 (57%)
Number of distinct spacers matched chromosome	1	0
Number of distinct spacers matched prophage	13	13
Number of distinct spacers matched own prophages	0	0

A spacer similar to the sequence of the host gene *trcF* was observed in MGAS2096 and MGAS9429 (Fig. S3). However, the spacer has a one-base pair difference with the *trcF* gene sequence and the *trcF* sequence is conserved among *S. pyogenes* strains as well as in *S. dysgalactiae*. This suggests that the spacer sequence, but not *trcF*, was mutated and has a relatively low impact on the host.

The CRISPR/Cas system is thought to provide the host bacteria with resistance against not only phages but also various mobile genetic elements [12]. It is known that several exogenous integrated conjugative elements (ICEs) are present in the *S. pyogenes* genome and the ICEs contain various genes such as antibacterial resistance genes [29]. Therefore, we investigated whether *S. pyogenes* CRISPR also contain spacers to counteract ICEs. In spite of the presence of 12 ICEs distributed in 13 sequenced strains, we could not find any spacers that showed similarity with the sequences of ICEs in *S. pyogenes* strains (data not shown).

Although all 13 *S. pyogenes* strains possess multiple prophages, five of them lack CRISPR/*cas* loci and the existing CRISPR have relatively few spacers. This implies that *S. pyogenes* CRISPR could not have functioned as an immunity system against invading phages. Instead, the spacer contents suggest that CRISPR appears to have functioned to inhibit phage insertions.

### Inverse correlation of *S. pyogenes* prophages and the number of spacers in a CRISPR locus

**Figure 3:**
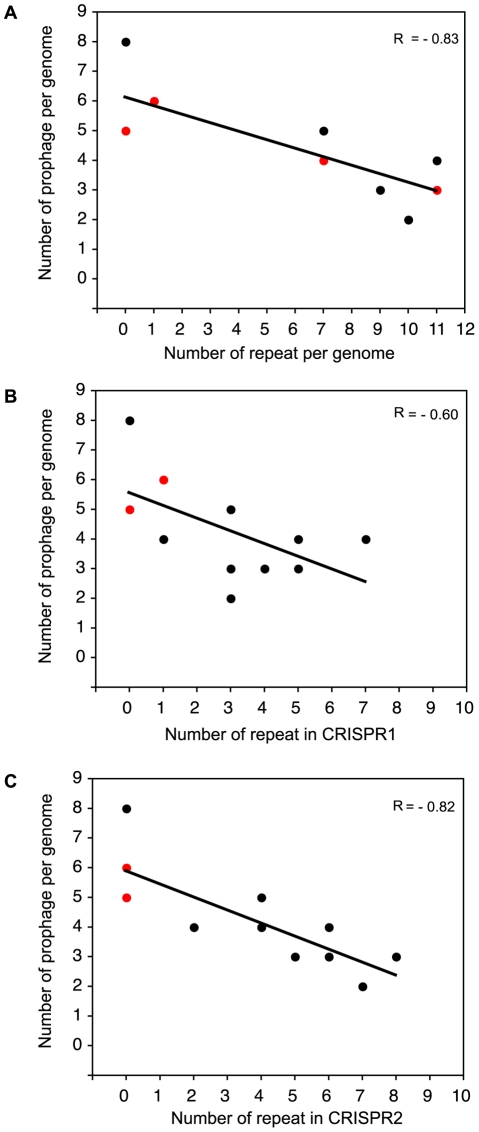
The relationship between the number of repeat sequences and prophages in *S. pyogenes*. (A), (B) and (C) show the number of prophages per genome versus the number of repeats per genome in CRISPR1 and CRISPR2, respectively. Red dots means overlapping of two dots.

To further confirm the function of *S. pyogenes* CRISPR, we investigated whether CRISPR-possessing strains have fewer prophages than those lacking CRISPR by a Wilcoxon rank sum test. We addressed the null hypothesis that there is no difference in the numbers of prophages between the CRISPR-positive and -negative strains and the null hypothesis could be rejected (*P*<0.01) indicating that the numbers of acquired prophages significantly differ between CRISPR-positive and -negative *S. pyogenes* strains. We next investigated whether a correlation exists between the number of spacers and prophages in each *S. pyogenes* genome. As shown Fig. 3A, we observed a clear inverse correlation between the total number of repeats per genome and the number of prophages within each genome (R = −0.83; *P*<0.001). These results strongly suggest that *S. pyogenes* CRISPR functions to inhibit phage incorporation into host genomes which depends on spacer number. Interestingly, although the correlation between the number of CRISPR2 repeats and the number of prophages was also clear (R = −0.82; *P*<0.001), the negative correlation between the number of CRISPR1 repeats and the number of prophages was relatively low (R = −0.60; *P*<0.05) (Fig. 3B, C), indicating that CRISPR2 may be more active than CRISPR1 in *S. pyogenes* genomes. Taken together, it was suggested that the absence of CRISPR/*cas* loci in five of the genomes and the low number of spacers in the seven strains with a functional CRISPR loci may explain the relatively high number of prophages in *S. pyogenes*.

### Insertion of phages into the terminal repeat-like sequence of *S. pyogenes* MGAS315

**Figure 4:**
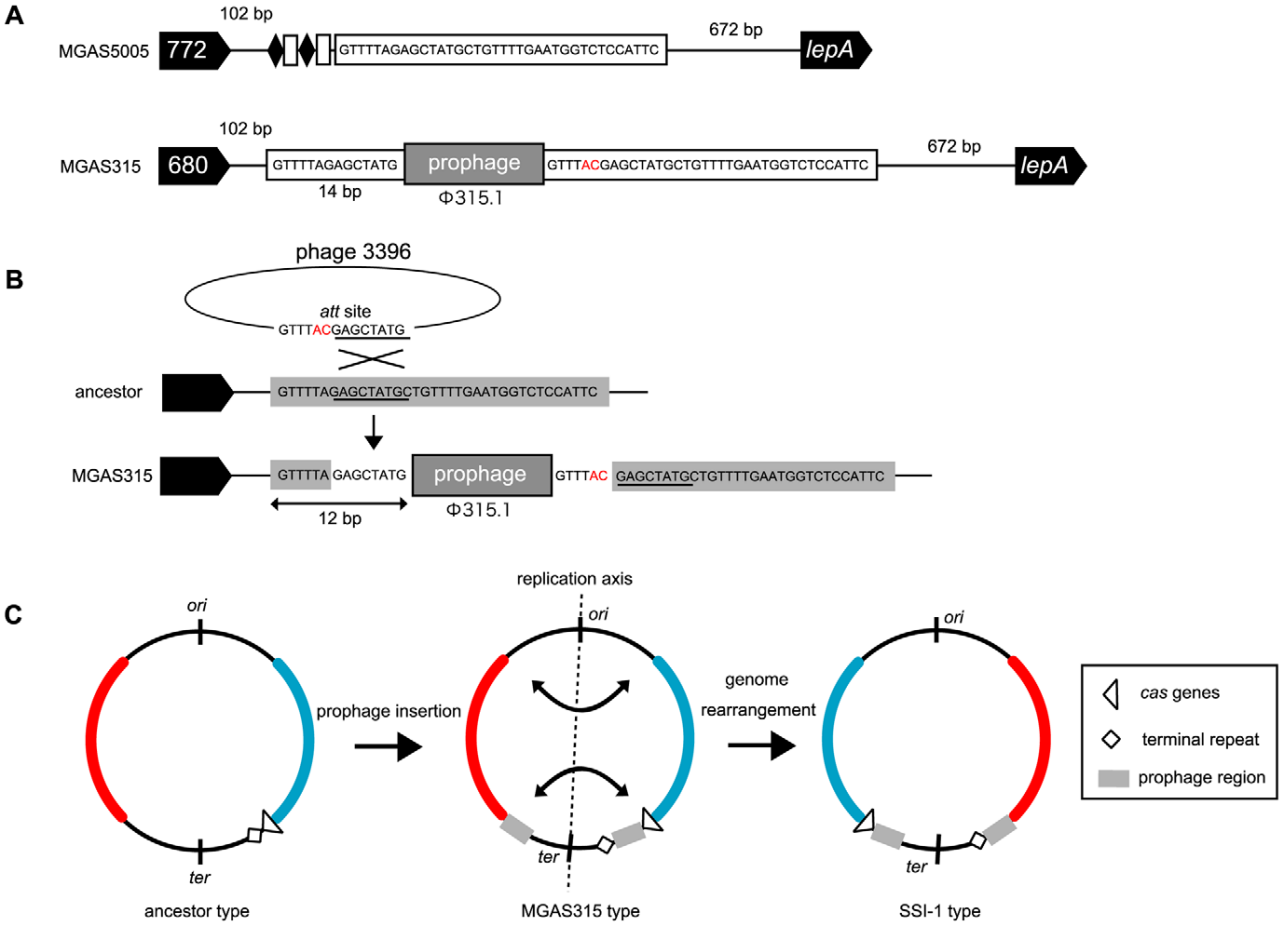
Phage insertion into the repeat sequences of MGAS315 and genome rearrangements. (A) The adjacent sequence of the Φ315.1 insertion site in the MGAS315 genome. (B) A scheme forΦ315.1 insertion into the repeat sequences of MGAS315. (C) Schematic diagram of the evolutionary relationship of MGAS315 and SSI-1 following phage insertion and genome rearrangement.

As described above, in MGAS315 *cas* genes are located in the same locus as that of the other CRISPR harboring strains and a terminal repeat-like sequence was found about 39.5 kb from the *cas* genes (Fig. S1B). In SSI-1, the terminal repeat-like sequence is located at the same position as MGAS315, and *cas* genes are located far away from the loci of *cas* genes in other strains. Since it was reported that the location of all CRISPR adjacent to the *cas* genes is required for their role as an acquired immunity system [11], it was suggested that the CRISPR/Cas system was not functional in the two strains. The prophage region (Φ 315.1) was found in between a terminal repeat-like sequence and *cas* genes of MGAS315 and SSI-1 also has a prophage (Φ SPsP5) just downstream of the *cas* genes (Fig. S2). From further detailed analysis of sequences around the prophage insertion site and between the terminal repeat-like sequence and *cas* genes, we found a portion of a terminal repeat sequence (14 bp in length) upstream of the prophage (Fig. 4A). The sequence of the prophage (Φ 315.1) in MGAS315 is highly similar to the sequence of Phage 3396 and these two phages were designated as the Φ315.1-like family by Davies [47]. We investigated the Phage 3396 genome and found that a region of the Phage 3396 genome is identical to an upper 12 bp of the terminal repeat-like sequence in MGAS315 and is the region for an *att* site (Fig. 4B). It is suggested, therefore, that the *att* site sequence can be used for recombination and enables phage genomes to be incorporated into the host MGAS315 genome. As shown Figure 4B, if recombination between the 14 bp sequence at the *att* site of the phage (GAGCTATG) and the homologous terminal repeat sequence (GAGCTATG) occurred, a combined sequence (GTTTTAGAGCTATG) is formed upstream of the prophage and a chimeric sequence of the former part of *att* site and the latter part of the repeat is produced downstream of the prophage (Fig. 4B). These observations could explain how the terminal repeat-like sequence of MGAS315, which is different by 2 bases from the typical terminal repeat sequence of CRISPR1, was produced after separation of the terminal sequence and *cas* genes by insertion of the phage into the MGAS315 genome.

We also compared the leader sequences among the strains and found that the leader sequence of MGAS315 was the same as those of other CRISPR1-harboring strains. This result suggests that the insertion of phages into the terminal repeat sequence of MGAS315 did not occur because of the impairment of the CRISPR system following mutation of the leader sequence.

### Derivative strain, SSI-1, from MGAS315 by large-scale genomic recombination

As with MGAS315, the position of the terminal repeat sequence of SSI-1 is separated from the *cas* genes and the distance in SSI-1 is 187 kbp further than that of MGAS315. The average of Single Nucleotide Polymorphism (SNP) per gene comparing MGAS315 and SSI-1 is 0.05% and high similarity is recognized between these genomes [48]. Nevertheless, the relative position of the CRISPR sequence and *cas* genes in the MGAS315 genome is clearly distinct from SSI-1. This can be explained by large genomic rearrangements which have occurred during evolution of the two strains [48]. Genomic rearrangement is a phenomenon whereby a genome region is exchanged between homologous sequences [49]. Although it was showed that large-scale genomic recombinations occurred in MGAS315 or SSI-1 [48], which is the ancestral strain has not been determined. In this study, we showed that MGAS315 was generated from the ancestral strain following the insertion of phage, suggesting that MGAS315 is the progenitor of SSI-1 (Fig. 4C).

### Distribution of prophages and CRISPR loci in other streptococci

To further examine the relationship between CRISPR/Cas systems and prophages in the genus Streptococcus, we expanded the analysis to an additional 35 bacterial sequenced streptococci. Prophage elements were widely distributed and observed in both CRISPR-harboring and CRISPR-lacking species (Fig. S4). To examine the impact of CRISPR on the acquisition of prophages for the genus of Streptococci, we evaluated whether CRISPR-possessing species have fewer prophages than those lacking CRISPR by a Wilcoxon rank sum test. We addressed the null hypothesis that there is no difference in the distributions of prophages between the CRISPR-positive and -negative species, and the null hypothesis could not be rejected (*P*>0.05). Similar results were obtained when the same analysis was performed with the 35 streptococci strains omitting the 13 *S. pyogenes* strains. Therefore, we could not conclude that CRISPR-lacking streptococci have acquired more prophages than CRISPR-harboring ones. However, it is possible that the influences of CRISPR on the acquisition of prophage vary among species. We then examined the distribution of prophages and CRISPR within each species though the number of sequenced strains per species was relatively small. *S. mutans*, *S. gordonii*, *S. sanguinis*, some *S. agalactiae*, and some *S. thermophilus* possess CRISPR loci and do not have any phage inserted into their genomes, whereas *S. mitis* and some strains of *S. pneumoniae* and *S. suis* do not have CRISPR locus and permitted insertion of phages into their genomes (Fig. S4). However, some *S. agalactiae*, *S. dysgalactiae*, *S. equi*, and *S. thermophilus* possess both CRISPR loci and prophage elements, and other *S. pneumoniae* strains have no prophage despite they lack the CRISPR locus (Fig. S4). These observations suggest that CRISPR may be involved in the inhibition of phage insertion even in streptococci other than *S. pyogenes* and the degree of contribution of CRISPR to the restriction of phage insertion varies among species. Further study is needed with more complete genome sequences to delineate more clearly the effects of CRISPR systems on genomic evolution.

## Discussion

*S. pyogenes* strains are well-known to induce a variety of diseases and the sequences of 13 strains suggested that the prophage regions contain variable virulence genes and confer pathogenic capacities [31]. In this study, we examined all 13 sequenced *S. pyogenes* strains and evaluated the relationship between CRISPR and the acquisition of prophages in *S. pyogenes*. We found that five of 13 *S. pyogenes* strains lacked CRISPR/*cas* loci and the existing CRISPR in seven strains have essentially complete repeat sequences and *cas* gene systems. Furthermore, the number of spacers was fewer than that of other CRISPR-harboring streptococci. As the number of spacers is a reliable indicator of CRISPR activity [43], the activities of *S. pyogenes* CRISPR may be lower than for other streptococcal CRISPR.

Spacer content and arrays suggested that most spacers were deleted and the acquisition of new spacers is an ongoing process. It is known that the Cas1 protein is involved in a spacer-acquisition step dependent upon its endonuclease activity [18], [50]. There is also a report that *S. mutans* UA159 has a truncating mutation in its *cas1* gene and its CRISPR contains only one spacer [42], supporting the importance of Cas1 in spacer acquisition. In our study, we could not find any apparent mutation in *S. pyogenes cas1*. Although it was recently reported that the divalent metal binding site of Cas1 are likely important for the function in *Pseudomonas aeruginosa* CRISPR [50], the *cas1* gene of *P. aeruginosa* varies greatly from that of *S. pyogenes* or other streptococci, and the active sites for streptococcal Cas1 activity have not yet been identified. So, we cannot exclude the possibility that *S. pyogenes* Cas proteins have undefined defect(s) such as a single amino-acid substitution in active site regions in some strains. In *S. thermophilus*, *csn2* (*cas7*) gene was shown to be involved in the spacer acquisition [11]. *csn2* is a member of the Nmeni subtype *cas* genes and widely harbored in Streptococcal genomes including *S. pyogenes*, and *S. pyogenes csn2* showed the high similarity with that of *S. thermophilus*. As well as the case of *cas1*, further detailed investigation of *cas* genes will be useful to determine whether the currently identified *S. pyogenes* Cas proteins have any defects.

The deletion of spacers is frequently observed in other bacteria, which is thought to be necessary to prevent over-inflation of the CRISPR locus [39], [51], [52]. The lacking of several spacers was observed among same M1 type *S. pyogenes* strains, indicating that spacer deletion frequently occurs in *S. pyogenes* CRISPR [35]. We also found evidence of frequent spacer deletion in *S. pyogenes* CRISPR. However, it is not clear whether such deletions occur actively or result from passive homologous recombination. Considering the irregularity of spacer deletion in *S. pyogenes*, the deletions may be a consequence of spontaneous homologous recombination events. Therefore, one of the reasons for the small number of *S. pyogenes* CRISPR spacers may be that homologous recombinations have frequently occurred and/or the *S. pyogenes* CRISPR/Cas system is unable to acquire new spacers as efficiently as other streptococcal CRISPR due to unknown effect(s).

Despite the low number of spacers in *S. pyogenes* CRISPR, spacer contents and the relationship between the number of repeats and prophages showed that *S. pyogenes* CRISPR has rejected the phage insertion. Interestingly, the inverse correlation between number of spacers and phages is significant for CRISPR2 (R = −0.82; Fig. 3C), and less so for CRISPR1 (R = −0.60; Fig. 3B). We found that larger portion of spacers in CRISPR1 (82.6%; 19/23) showed high similarity to known sequences, whereas smaller portion of that in CRISPR2 (64.7%; 22/34) matched with known sequences. Among these spacers, 21.1% (4/19; in CRISPR1) and 45.5% (10/22; in CRISPR2) of spacers match prophages with >95% identity but not 100%. Since phages specifically mutated the proto-spacer to overcome CRISPR/Cas immunity [19], it may be suggested that spacers in CRISPR1 are acquired more recently than that in CRISPR2. This may be the reason why CRISPR1 showed less inverse correlation compared to CRISPR2.

Recently, there was a report that transcription of the *cas* genes and some CRISPR arrays is repressed by heat-stable nucleoid-structuring (H-NS) proteins in *Escherichia coli* [53], suggesting the existence of a CRISPR regulation system. Transmittable phages and plasmids contain unique genetic elements which could confer novel characteristics on the recipients. In addition, these characteristic contributions are thought to be important for host environmental adaptation. For example, lysogenic infection of *P. aeruginosa* with bacteriophage DMS3 inhibits biofilm formation and swarming motility and this inhibition requires the CRISPR region [54]. Based on this report, Papenfort also suggested that some pathogens might adapt CRISPR activity to control prophage-encoded genes for virulence [55]. More recently, it was reported that Enterococci have acquired antibiotic resistant genes through the loss of CRISPR/*cas* loci demonstrating an additional role for CRISPR/*cas* in HGT [37]. In the case of *S. pyogenes*, despite prophage-encoded genes providing important characteristics to the organism, CRISPR/*cas* is present in their genomes and appears to function in inhibiting phage insertions. This raises the possibility that *S. pyogenes* might have evolved its CRISPR activity for incorporation of beneficial phages into its own genome.

The CRISPR/Cas system was reported to have a potential for influencing genome-scale evolution involving spacers which are homologous to chromosomal genes in *Pelobacter carbinolicus* [56]. In *S. pyogenes* CRISPR, one spacer in MGAS2096 and MGAS9429 was similar to the sequence within *trcF*, a transcription repair coupling factor gene of *S. pyogenes*. TrcF has been well studied in *E. coli* and is known to be involved in the transcription coupled repair system for DNA which operates in tandem with transcription [57]. Therefore, there it is possible that the spacers could influence DNA repair systems. However, for *S. pyogenes*, the spacer sequence has a one-base difference from the *trcF* gene sequence and this later sequence is conserved among *S. pyogenes* strains as well as in *S. dysgalactiae*. This suggests that it has a relatively low impact on the host. To definitely determine the effects of the spacer on the host, further experimental studies will be necessary.

As for MGAS315 and SSI-1, because these strains have almost completely conserved genome sequences, it was difficult to determine which is the more primitive strain. In this study, we suggested that MGAS315 is the ancestral strain of SSI-1. Although the insertion of phages into the repeat sequences may be a random event, we propose the possibility that this event is a novel anti-CRISPR mechanisms which allow phages to subvert CRISPR antagonism and facilitate entry of more phages into the host genome. Of note, CRISPR have been identified within two prophages in *Clostridium difficile* [58]. When the prophages inserted into the CRISPR are deleted due to homologous recombination between the repeat sequences that are located external to the prophages, CRISPR-containing phages are produced. Therefore, the invasion of phages into the repeat sequences may be required not only for inactivation of the CRISPR/Cas system but also for the acquisition of CRISPR. However, the identification of CRISPR in prophage regions is still somewhat limited so additional comprehensive research may provide further interesting findings regarding the relationship between phages and CRISPR.

As a result of the analysis of an additional 35 streptococcal genomes, the influences of CRISPR loci on the distributions of prophages appeared to be species dependent. This could be explained by the presence of other anti-phage systems or unknown environmental effects. If these species have additional strict self-defense systems against invading phages or their habitats contain fewer phages, the CRISPR/*cas* locus may be unnecessary. Therefore, even in the same genus, the contribution of CRISPR to phage sensitivity seems to depend on the species and bacteria might have evolved their intrinsic self-defense systems depending on their environments. Prophage identification using prophage prediction tools such as Prophinder [59] is principally based on similarity searches, gene annotation and detection of conserved pairs of genes found in phage genomes. Moreover, where similar prophages are inserted into one genome, it is very difficult to identify the actual number of prophages [59]. Therefore, to understand the relationship between the CRISPR/Cas system and prophages, more complete genome sequences are required. We also showed the importance of comparative genome analysis in CRISPR research in previous studies [37], [43]. However, experimental studies will also be indispensable to verify our findings.

The involvement of CRISPR in bacterial adaptation to their environments is suggested in enterococci which lack endogenous CRISPR/*cas* loci and can obtain new antibiotic resistance genes in antibiotic treated environments [37]. Likewise, *S. pyogenes* might have evolved limited CRISPR/Cas activity to enhance the acquisition of virulence genes, and this phenomenon might have contributed to the diverse strain-specific pathogenicities observed in this important pathogen. More generally, the absence of CRISPR could be one important survival strategy for human pathogens.

## Materials and Methods

### Genomes

The information for the complete genome sequences of streptococcal strains used in this study was derived from the search results of the Gold genome online database (http://www.genomesonline.org/) as complete sequences as of October 20, 2010. These data include 13 strains of *S. pyogenes* (accession no. NC_002737, NC_007297, NC_003485, NC_007296, NC_004606, NC_011375, NC_006086, NC_009332, NC_008022, NC_008024, NC_008023, NC_004070, NC_008021), three strains of *S. agalactiae* (NC_007432, NC_004116, NC_004368), one strain of *S. dysgalactiae* (NC_012891), three strains of *S. equi* (NC_012471, NC_012470, NC_011134), one strain of *S. gallolyticus* (NC_013798), one strain of *S. gordonii* (NC_009785), one strain of *S. mitis* (NC_013853), two strains of *S. mutans* (NC_013928, NC_004350), 12 strains of *S. pneumoniae* (NC_011900, NC_012468, NC_010582, NC_008533, NC_011072, NC_010380, NC_012466, NC_012467, NC_003098, NC_012469, NC_014251, NC_003028), one strain of *S. sanguinis* (NC_009009), six strains of *S. suis* (NC_009442, NC_009443, NC_012926, CP_000837, NC_012925, NC_012924), three strains of *S. thermophilus* (NC_006449, NC_008532, NC_006448), and one strain of *S. uberis* (NC_012004). Each complete gene sequence was obtained from the NCBI (http://www.ncbi.nlm.gov) database.

### CRISPR analysis

The CRISPR candidates were obtained with CRISPRfinder (http://crispr.u-psud.fr/Server/) [60]. The CRISPR candidates were confirmed manually by examining their adjacent sequences [34]. We added the CRISPR data for *S. mutans* and *S. thermophilus* from previous reports [20], [27], [42]. The classification of repeat clusters was based upon a previous study [32]. To investigate proto-spacers, the nucleotide sequence database was queried with each of the CRISPR spacers of *S. pyogenes* using Blastn of NCBI with default parameters for short input sequences [61]. Amino acid and nucleic acid sequence alignments were generated with ClustalW in DDBJ (http://clustalw.ddbj.nig.ac.jp/top-j.html) [62].

### Prophage identification

To identify the distribution of prophages in streptococci, we used Prophinder (http://aclame.ulb.ac.be/Tools/Prophinder/) [59]. We submitted Genbank files of genome data obtained from NCBI to a query system of Prophinder and generated the results for prophage prediction.

### Analysis of streptococcal CRISPR and prophage distribution

To test whether the numbers of acquired prophage elements differ in the presence or absence of CRISPR/loci, we performed the nonparameric Wilcoxon rank sum test.

### Phylogenetic tree of *S. pyogenes*

Thirteen concatenated sequences were obtained from MLST.net [63] and aligned with the ClustalW-2.0.12 Software [62]. The MEGA4 program [64] was used to estimate nucleotide diversity and evolutionary distances as well as to build phylogenetic trees by the neighbor-joining method [65] using the Jukes-Cantor distances [66], which were selected for nucleotide substitutions using jModelTest, version 0.1.1 [67]. The reliability of clustering patterns in the phylogenetic trees was assessed by bootstrapping [68] and 1000 bootstrap pseudo-samples were used. Before conducting the phylogenetic analysis, we tested for recombination using the PHI test as implemented in the SplitsTree4 program [69] and no recombination events were detected (cutoff value: *P*<0.05). The data were mid-point-rooted and images were created using FigTree v1.3.1 [70].

## Supporting Information

Figure S1 Position of the CRISPR/*cas* and exogenous elements loci in the chromosome. (A) The position of prophages, ICEs, and CRISPR/*cas* loci were shown. The position of prophages and ICEs were followed by Beres et al [28]. CRISPRs are indicated with diamond shapes, *cas* genes set with rectangles, prophages with triangles, and ICEs with circles. Stacked shapes indicate a common insertion site. Elements are color-coded to indicate the source strain. (B) Enlarged figure of the position of *cas* genes and terminal repeat-like sequence in MGAS315 and SSI-1 was shown.
(PDF)

Figure S2 *S. pyogenes* CRISPR locus overview. Nmeni *cas* subtype is characterized by the presence of 4 successive genes; *csn1*, *cas1*, *cas2*, and *csn2*. Dvulg *cas* subtype is characterized by 7 successive genes; *cas3*, *cas5*, *csd1*, *cds2*, *cas4*, *cas1*, and *cas2*. Repeat-spacer array are shown as white boxes. Same or homologous genes are represented by identical color boxes.
(PDF)

Figure S3 Graphic representation of spacers matched a known sequence. Repeats are not included. The spacers matched a known sequence are represented with colored box. The spacers does not match a known sequence are represented with white box. Names of prophage or bacterial chromosome gene sequences that are matched with the spacer were shown under the boxed (perfect identity: black character, >95% identity: colored character).
(PDF)

Figure S4 CRISPR and prophage distribution in all sequenced Streptococci. All 48 sequenced streptococci are listed. Possessing prophage is shown in blue, and CRISPR locus presence is shown in red. The number of prophage, CRISPR loci or repeats are shown.
(PDF)

Table S1 Repeat sequences of *S. pyogenes* CRISPR.
(PDF)

Table S2 Characteristics of CRISPR loci of Streptococci.
(PDF)
