# Supplementary material for: CRISPR Inhibition of Prophage Acquisition in Streptococcus pyogenes
Source: PLoS One. 2011 May 6;6(5):e19543. doi: 10.1371/journal.pone.0019543 (PMC3089615; doi:10.1371/journal.pone.0019543)
Supplement: Figure S1 — Position of the CRISPR/cas and exogenous elements loci in the chromosome. (A) The position of prophages, ICEs, and CRISPR/cas loci were shown. The position of prophages and ICEs were followed by Beres et al [28]. CRISPRs are indicated with diamond shapes, cas genes set with rectangles, prophages with triangles, and ICEs with circles. Stacked shapes indicate a common insertion site. Elements are color-coded to indicate the source strain. (B) Enlarged figure of the position of cas genes and terminal repeat-like sequence in MGAS315 and SSI-1 was shown. (PDF) [file pone.0019543.s001.pdf]

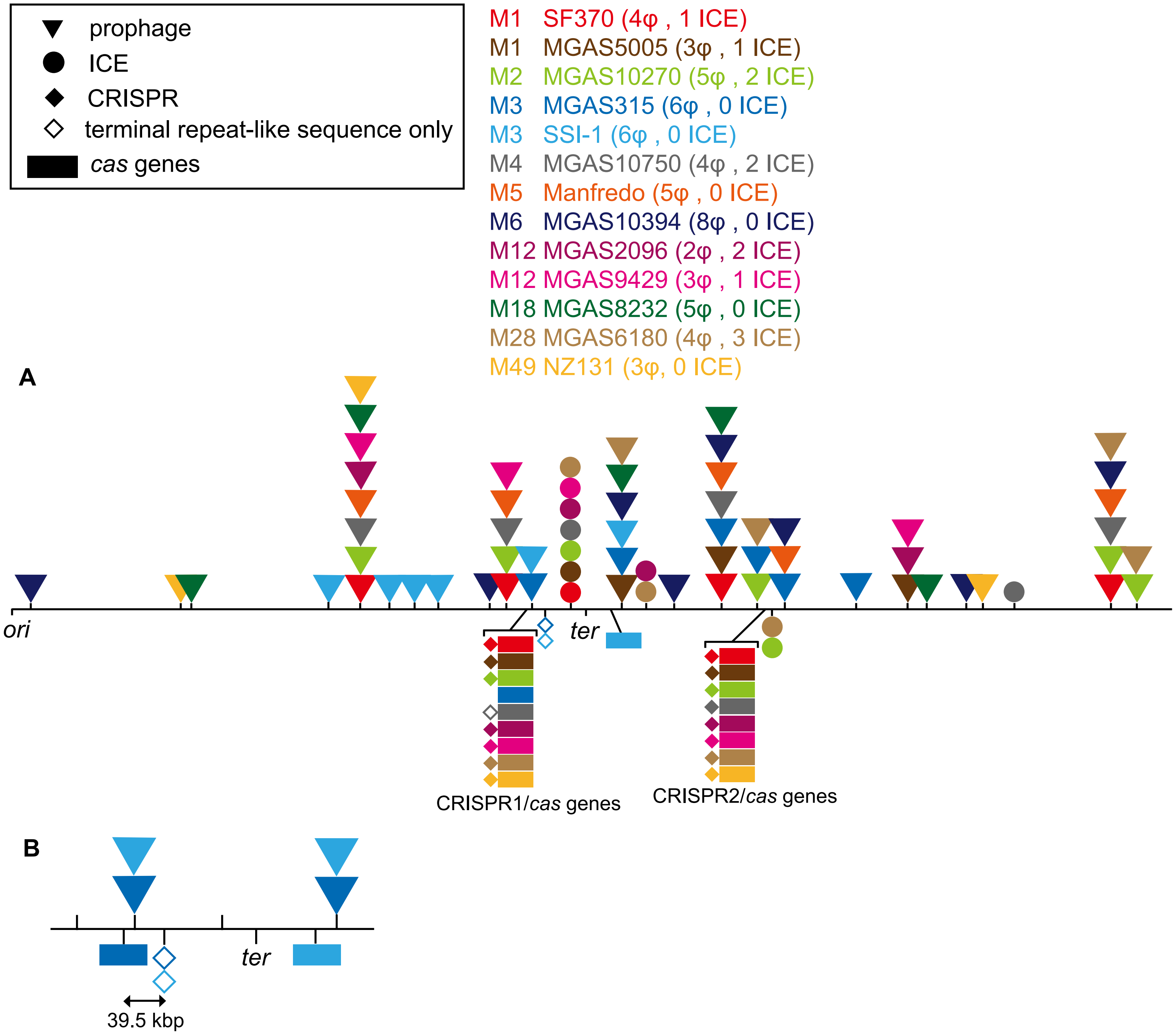

prophage
ICE
CRISPR
terminal repeat-like sequence only
cas genes
M1 SF370 (4φ , 1 ICE)
M1 MGAS5005 (3φ , 1 ICE)
M2 MGAS10270 (5φ , 2 ICE)
M3 MGAS315 (6φ , 0 ICE)
M3 SSI-1 (6φ , 0 ICE)
M4 MGAS10750 (4φ , 2 ICE)
M5 Manfredo (5φ , 0 ICE)
M6 MGAS10394 (8φ , 0 ICE)
M12 MGAS2096 (2φ , 2 ICE)
M12 MGAS9429 (3φ , 1 ICE)
M18 MGAS8232 (5φ , 0 ICE)
M28 MGAS6180 (4φ , 3 ICE)
M49 NZ131 (3φ, 0 ICE)
A
ori
ter
CRISPR1/cas genes
CRISPR2/cas genes
B
ter
39.5 kbp
