# Supplementary figures and images for: CRISPR Inhibition of Prophage Acquisition in Streptococcus pyogenes
Source: PLoS One. 2011 May 6;6(5):e19543. doi: 10.1371/journal.pone.0019543 (PMC3089615; doi:10.1371/journal.pone.0019543)

Nmeni cas subtype

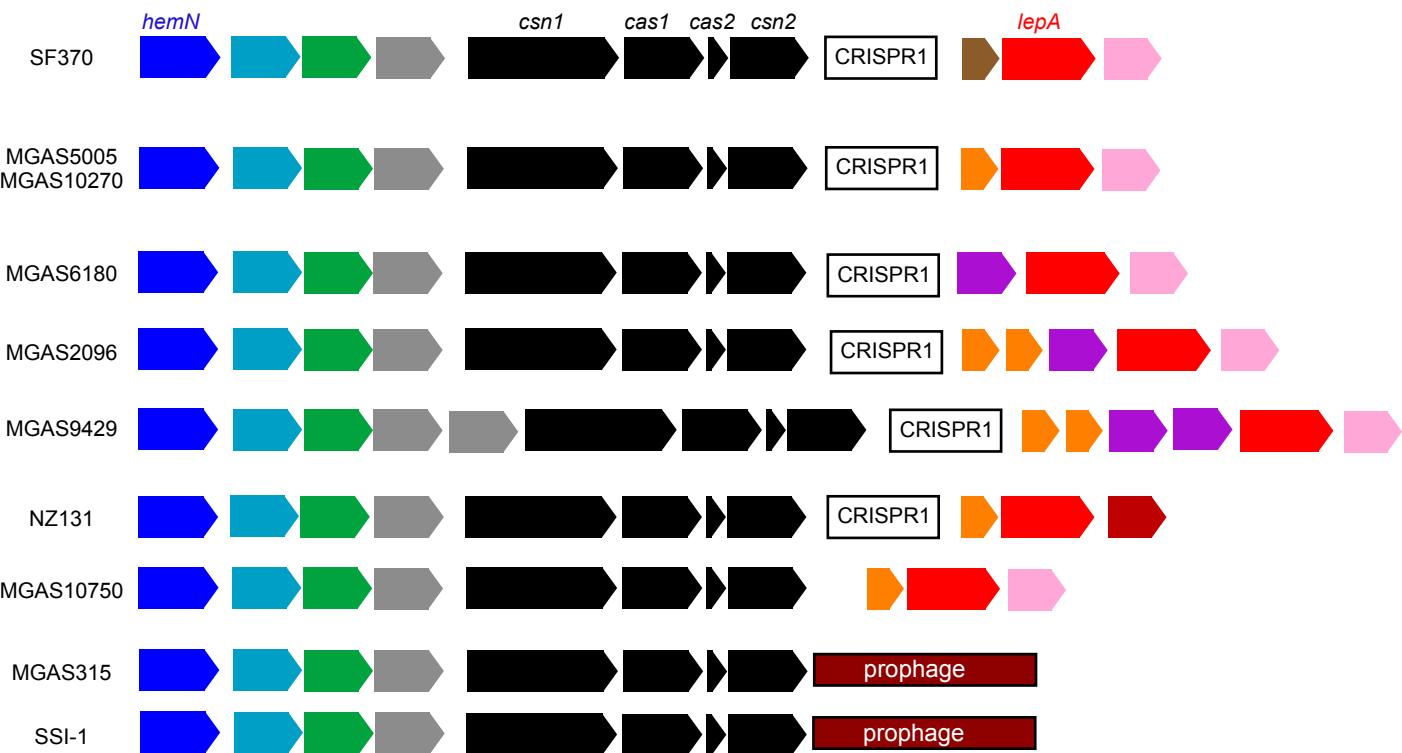

Dvulg cas subtype

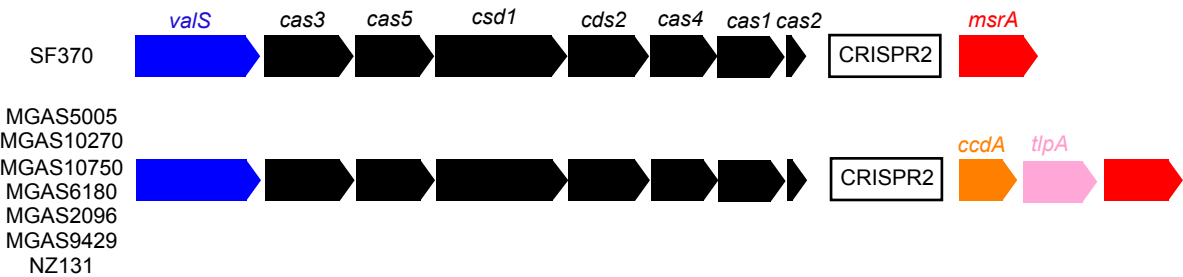

Supplement: Figure S2 — S. pyogenes CRISPR locus overview. Nmeni cas subtype is characterized by the presence of 4 successive genes; csn1, cas1, cas2, and csn2. Dvulg cas subtype is characterized by 7 successive genes; cas3, cas5, csd1, cds2, cas4, cas1, and cas2. Repeat-spacer array are shown as white boxes. Same or homologous genes are represented by identical color boxes. (PDF) [file pone.0019543.s002.pdf]

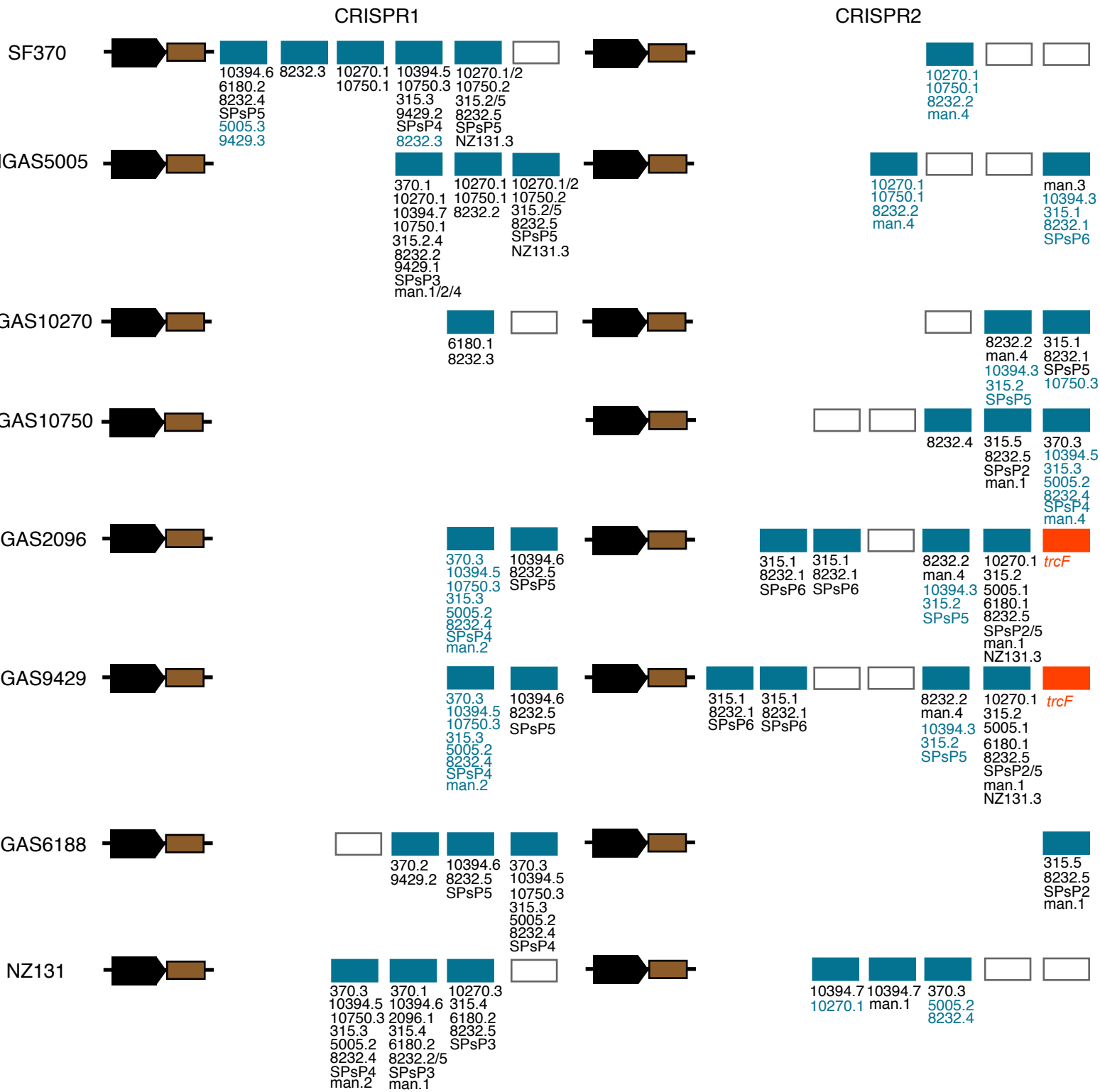

Supplement: Figure S3 — Graphic representation of spacers matched a known sequence. Repeats are not included. The spacers matched a known sequence are represented with colored box. The spacers does not match a known sequence are represented with white box. Names of prophage or bacterial chromosome gene sequences that are matched with the spacer were shown under the boxed (perfect identity: black character, >95% identity: colored character). (PDF) [file pone.0019543.s003.pdf]
