# Supplementary material for: CRISPR Inhibition of Prophage Acquisition in Streptococcus pyogenes
Source: PLoS One. 2011 May 6;6(5):e19543. doi: 10.1371/journal.pone.0019543 (PMC3089615; doi:10.1371/journal.pone.0019543)
Supplement: Figure S4 — CRISPR and prophage distribution in all sequenced Streptococci. All 48 sequenced streptococci are listed. Possessing prophage is shown in blue, and CRISPR locus presence is shown in red. The number of prophage, CRISPR loci or repeats are shown. (PDF) [file pone.0019543.s004.pdf]

	Prophage	CRISPR	Repeats
Streptococcus agalactiae 2603V/R	3	1	25
Streptococcus agalactiae A909	3	1	15
Streptococcus agalactiae NEM316	3	1	14
Streptococcus dysgalactiae equisimilis GGS_124	2	1	19
Streptococcus equi equi 4047	4	0	0
Streptococcus equi zooepidemicus H70	1	1	19
Streptococcus equi zooepidemicus MGCS10565	1	1	28
Streptococcus gallolyticus UCN34	1	2	29
Streptococcus gordonii Challis CH1	0	1	27
Streptococcus mitis B6	1	0	0
Streptococcus mutans NN2025	0	2	89
Streptococcus mutans UA159	0	2	6
Streptococcus pneumoniae ATCC700669	1	0	0
Streptococcus pneumoniae 70585	2	0	0
Streptococcus pneumoniae CGSP14	0	0	0
Streptococcus pneumoniae D3	0	0	0
Streptococcus pneumoniae G54	0	0	0
Streptococcus pneumoniae Hungary 19A-6	4	0	0
Streptococcus pneumoniae JJA	2	0	0
Streptococcus pneumoniae P1031	2	0	0
Streptococcus pneumoniae R6	0	0	0
Streptococcus pneumoniae Taiwan19F-14	3	0	0
Streptococcus pneumoniae TCH8431/19A	3	0	0
Streptococcus pneumoniae TIGR4	0	0	0
Streptococcus pyogenes SF370	4	2	11
Streptococcus pyogenes MGAS5005	3	2	9
Streptococcus pyogenes MGAS8232	6	0	0
Streptococcus pyogenes MGAS6180	4	2	7
Streptococcus pyogenes SSI-1	6	0	1
Streptococcus pyogenes NZ131	3	2	9
Streptococcus pyogenes MGAS10394	8	0	0
Streptococcus pyogenes Manfredo	5	0	0
Streptococcus pyogenes MGAS10270	5	2	7
Streptococcus pyogenes MGAS10750	4	2	7
Streptococcus pyogenes MGAS2096	2	2	10
Streptococcus pyogenes MGAS9429	3	2	11
Streptococcus pyogenes MGAS315	6	0	1
Streptococcus sanguinis SK36	0	3	42
Streptococcus suis 05ZYH33	0	0	0
Streptococcus suis 98HAH33	1	0	0
Streptococcus suis BM407	1	0	0
Streptococcus suis GZ1	1	0	0
Streptococcus suis P1/7	1	0	0
Streptococcus suis SC84	1	0	0
Streptococcus thermophilus CNRZ 1066	0	1	42
Streptococcus thermophilus LMD-9	3	3	30
Streptococcus thermophilus LMG18311	0	3	43
Streptococcus uberis 04140J	1	0	0
