# Supplementary material for: CRISPR Inhibition of Prophage Acquisition in Streptococcus pyogenes
Source: PLoS One. 2011 May 6;6(5):e19543. doi: 10.1371/journal.pone.0019543 (PMC3089615; doi:10.1371/journal.pone.0019543)
Supplement: Table S1 — Repeat sequences of S. pyogenes CRISPR. (PDF) [file pone.0019543.s005.pdf]

Table S1. Repeat sequences of *S. pyogenes* CRISPR.

CRISPR	Strain	Typical repeat sequence	Terminal repeat sequence[a]
CRISPR1	SF370	GTTTTAGAGCTATGCTGTTTTGAATGGTCCCAAAAC	GTTTTAGAGCTATGCTGTTTTGAATGGTCTCCATTC
	MGAS5005	GTTTTAGAGCTATGCTGTTTTGAATGGTCCCAAAAC	GTTTTAGAGCTATGCTGTTTTGAATGGTCTCCATTC
	MGAS10270	GTTTTAGAGCTATGCTGTTTTGAATGGTCCCAAAAC	GTTTTAGAGCTATGCTGTTTTGAATGGTCTCCATTC
	MGAS315	- [b]	GTTT**AC**GAGCTATGCTGTTTTGAATGGTCTCCATTC
	SSI-1	- [b]	GTTT**AC**GAGCTATGCTGTTTTGAATGGTCTCCATTC
	MGAS10750	- [b]	GTTTTAGAGCTATGCTGTTTTGA**G**TGGTCTCCATTC
	MGAS2096	GTTTTAGAGCTATGCTGTTTTGAATGGTCCCAAAAC	GTTTTAGAGCTATGCTGTTTTGAATGGTCTCCATTC
	MGAS9429	GTTTTAGAGCTATGCTGTTTTGAATGGTCCCAAAAC	GTTTTAGAGCTATGCTGTTTTGAATGGTCTCCATTC
	MGAS6180	GTTTTAGAGCTATGCTGTTTTGAATGGTCCCAAAAC	GTTTTAGAGCTATGCTGTTTTGAATGGTCTCCATTC
	NZ131	GTTTTAGAGCTATGCTGTTTTGAATGGTCCCAAAAC	GTTTTAGAGCTATGCTGT**C**TTGAATGGTCTCCATTC
CRISPR2	SF370	ATTTCAATCCACTCACCCATGAAGGGTGAGAC	CTTTCAATCCACTCACCCATGAAGGGTGAGAC
	MGAS5005	ATTTCAATCCACTCACCCATGAAGGGTGAGAC	CTTTCAATCCACTCACCCATGAAGGGTGAGAC
	MGAS10270	ATTTCAATCCACTCACCCATGAAGGGTGAGAC	CTTTCAATCCACTCACCCATGAAGGGTGAGAC
	MGAS10750	ATTTCAATCCACTCACCCATGAAGGGTGAGAC	CTTTCAATCCACTCACCCATGAAGGGTGAGAC
	MGAS2096	ATTTCAATCCACTCACCCATGAAGGGTGAGAC	CTTTCAATCCACTCACCCATGAAGGGTGAGAC
	MGAS9429	ATTTCAATCCACTCACCCATGAAGGGTGAGAC	CTTTCAATCCACTCACCCATGAAGGGTGAGAC
	MGAS6180	ATTTCAATCCACTCACCCATGAAGGGTGAGAC	CTTTCAATCCACTCACCCATGAAGGGTGAGAC
	NZ131	ATTTCAATCCACTCACCCATGAAGGGTGAGAC	CTTTCAATCCACTCACCCATGAAGGGTGAGAC

[a] Bold characters are different bases from the most frequent sequence.

[b] The absence of typical repeat sequence is shown.
