# Supplementary material for: CRISPR Inhibition of Prophage Acquisition in Streptococcus pyogenes
Source: PLoS One. 2011 May 6;6(5):e19543. doi: 10.1371/journal.pone.0019543 (PMC3089615; doi:10.1371/journal.pone.0019543)
Supplement: Table S2 — Characteristics of CRISPR loci of Streptococci. (PDF) [file pone.0019543.s006.pdf]

Table S2. Characteristics of CRISPR loci of Streptococci.

Species	CRISPR	Repeat cluster [a]	Repeat size (bp)	Repeat number per locus	Repeats number per genome	Spacer size (bp)	Distribution (%)[c]
S. agalactiae	CRISPR1	10	36	18.0 (14-25) [b]	18.0	30 (29-31) [b]	100 (3/3)
S. disgalactiae	CRISPR1	10	36	19	19	30	100 (1/1)
S. equi	CRISPR1	3	32	19	23.5	33 (33-37) [b]	33.3 (1/3)
	CRISPR2	3	32	10		35 (33-35)	33.3 (1/3)
	CRISPR3	10	36	18		30	33.3 (1/3)
S. gallolyticus	CRISPR1	10	36	14.5 (13-16) [b]	29	30 (29-30)[b]	100 (1/1)
S. gordonii	CRISPR1	10	36	27	27	30 (29-31) [b]	100 (1/1)
S. mutans	CRISPR1	10	36	19.4 (4-70)[b]	20.2	30 (28-31) [b]	70.4 (19/27)
	CRISPR2	3	32	18.2 (2-51)[b]		34 (33-36) [b]	33.3 (9/27)
S. pyogenes	CRISPR1	10	36	3.3 (1-7)[b]	6.6	30 (30-31) [b]	76.9 (10/13)
	CRISPR2	3	32	5.2 (2-8)[b]		35 (33-36)[b]	61.5 (8/13)
S. sanguinis	CRISPR1	8	29	14 (2-21)[b]	42	43 (41-47)[b]	100 (1/1)
S. thermophilus	CRISPR1	10	36	23.9 (3-52)[b]	36	30 (29-32)[b]	100 (124/124)
	CRISPR2	8	36	3.9 (1-9)[b]		36	90.8 (59/65)
	CRISPR3	16	36	14.2 (1-30)[b]		30 (30-32)[b]	80.3 (53/66)

[a] Repeat clusters were defined according to Kunin et al [32].

[b] (min-max)

[c] The rate of CRISPR-harboring strains was shown. Addition to the information of sequenced strains in this study, we added the data of *S. mutans* and *S. thermophilus* [20, 42].
